# Supplementary material for: New Phocoenamicin and Maklamicin Analogues from Cultures of Three Marine-Derived Micromonospora Strains
Source: Mar Drugs. 2023 Aug 7;21(8):443. doi: 10.3390/md21080443 (PMC10455904; doi:10.3390/md21080443)
Supplement: Supplementary file 1 [file marinedrugs-21-00443-s001.zip › marinedrugs-2519368-supplementary.pdf]

New Phocoenamicin and Maklamicin Analogues from Cultures of Three Marine-Derived *Micromonospora* Strains

Maria Kokkini [1,*], Daniel Oves-Costales [1], Pilar Sánchez [1], Ángeles Melguizo [1], Thomas A. Mackenzie [1], Mercedes Pérez-Bonilla [1], Jesús Martín [1], Arianna Giusti [2], Peter de Witte [2], Francisca Vicente [1], Olga Genilloud [1] and Fernando Reyes [1,*]

[1]Fundación MEDINA, Centro de Excelencia en Investigación de Medicamentos Innovadores en Andalucía, Parque Tecnológico Ciencias de la Salud, Avda. del Conocimiento 34, Armilla, 18016 Granada, Spain

[2] Laboratory for Molecular Biodiscovery, Department of Pharmaceutical and Pharmacological Sciences, University of Leuven, O & N II Herestraat 49-box 824, 3000 Leuven, Belgium

*Correspondence: maria.kokkini@medinaandalucia.es (M.K.); fernando.reyes@medinaandalucia.es (F.R.); Tel.: +34-958-993-965 (F.R.)

Contents

Figure S1. UV spectrum of compound **1**.
Figure S2. ESI-TOF spectrum of compound **1**.
Figure S3. ^{1}H NMR (methanol-*d*$_4$, 500 MHz) spectrum of compound **1**.
Figure S4. ^{13}C NMR (methanol-*d*$_4$, 125 MHz) spectrum of compound **1**.
Figure S5. HSQC spectrum of compound **1**.
Figure S6. COSY spectrum of compound **1**.
Figure S7. HMBC spectrum of compound **1**.
Figure S8. NOESY spectrum of compound **1**.
Figure S9. UV spectrum of compound **2**.
Figure S10. ESI-TOF spectrum of compound **2**.
Figure S11. ^{1}H NMR (methanol-*d*$_4$, 500 MHz) spectrum of compound **2**.
Figure S12. ^{13}C NMR (methanol-*d*$_4$, 125 MHz) spectrum of compound **2**.
Figure S13. HSQC spectrum of compound **2**.
Figure S14. COSY spectrum of compound **2**.
Figure S15. HMBC spectrum of compound **2**.
Figure S16. NOESY spectrum of compound **2**.
Figure S17. UV spectrum of compound **6**.
Figure S18. ESI-TOF spectrum of compound **6**.
Figure S19. ^{1}H NMR (methanol-*d*$_4$, 500 MHz) spectrum of compound **6**.
Figure S20. ^{13}C NMR (methanol-*d*$_4$, 125 MHz) spectrum of compound **6**.
Figure S21. HSQC spectrum of compound **6**.
Figure S22. COSY spectrum of compound **6**.
Figure S23. HMBC spectrum of compound **6**.
Figure S24. ROESY spectrum of compound **6**.
Figure S25. Key NOESY correlations observed in phocoenamicin E (**2**).
Figure S26. Key ROESY correlations observed in maklamicin B (**6**).

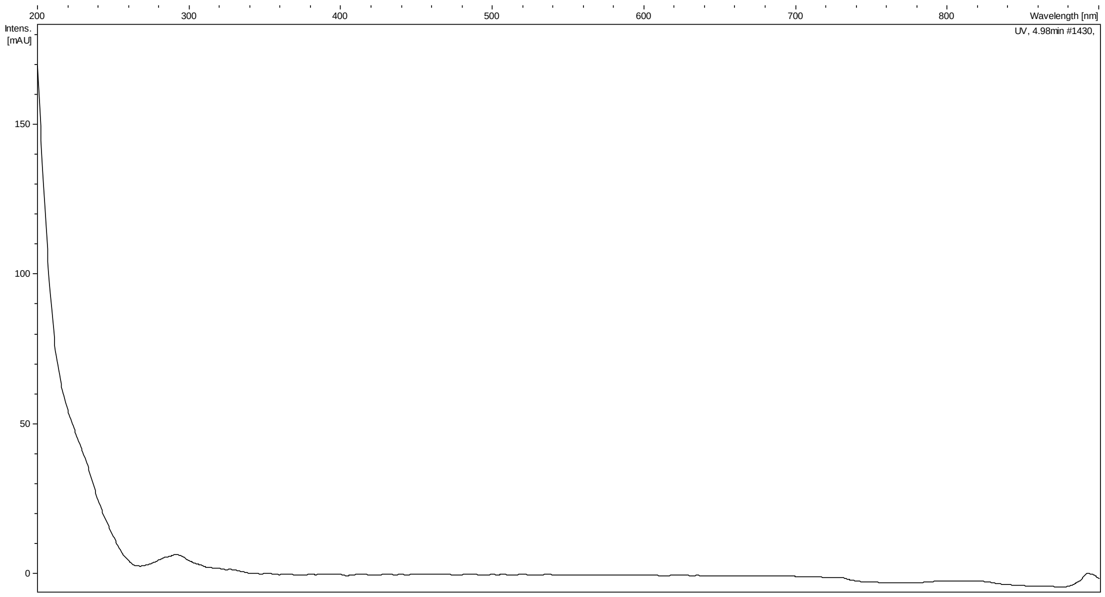

Figure S1. UV spectrum of compound **1**.

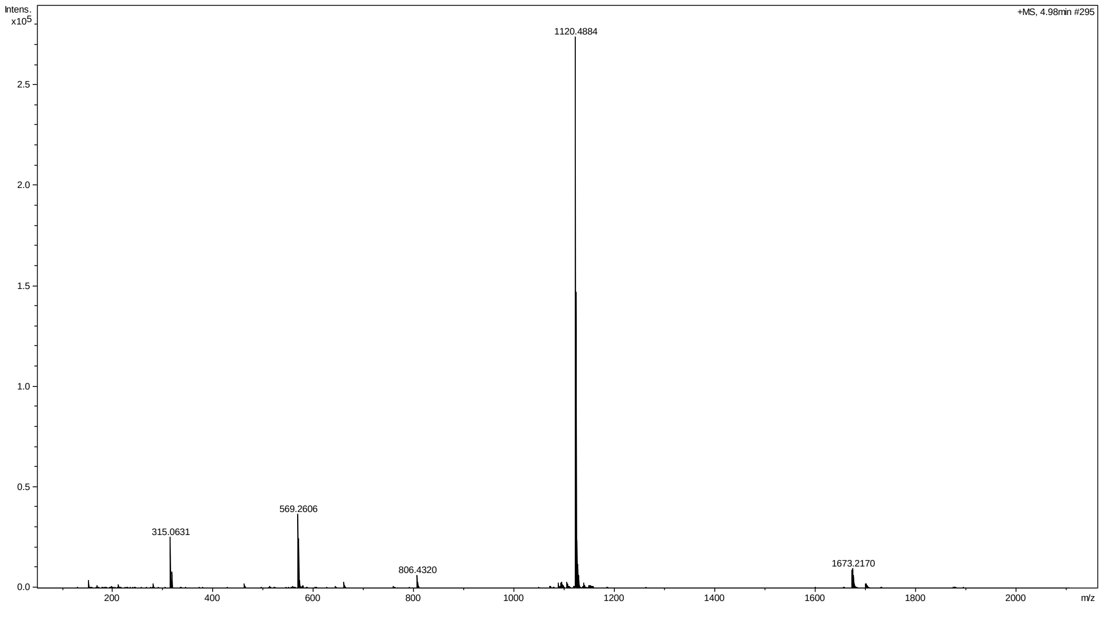

Figure S2. ESI-TOF spectrum of compound **1**.

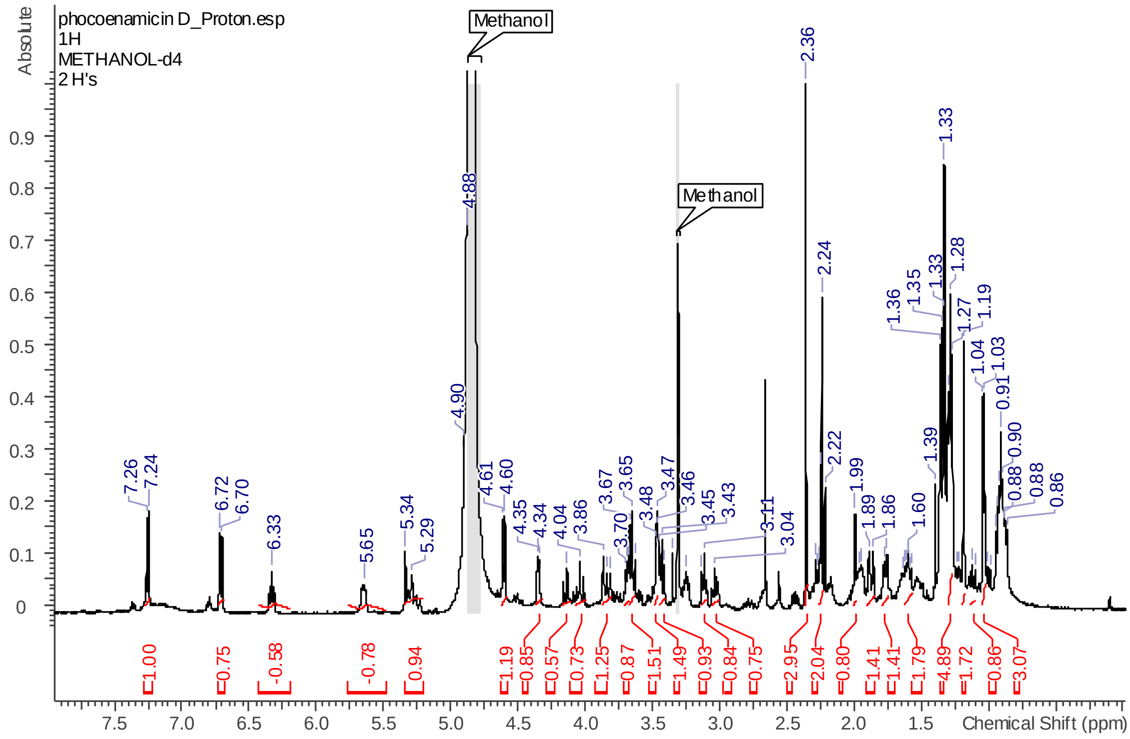

Figure S3. ^{1}H NMR (methanol-d_4, 500 MHz) spectrum of compound **1**.

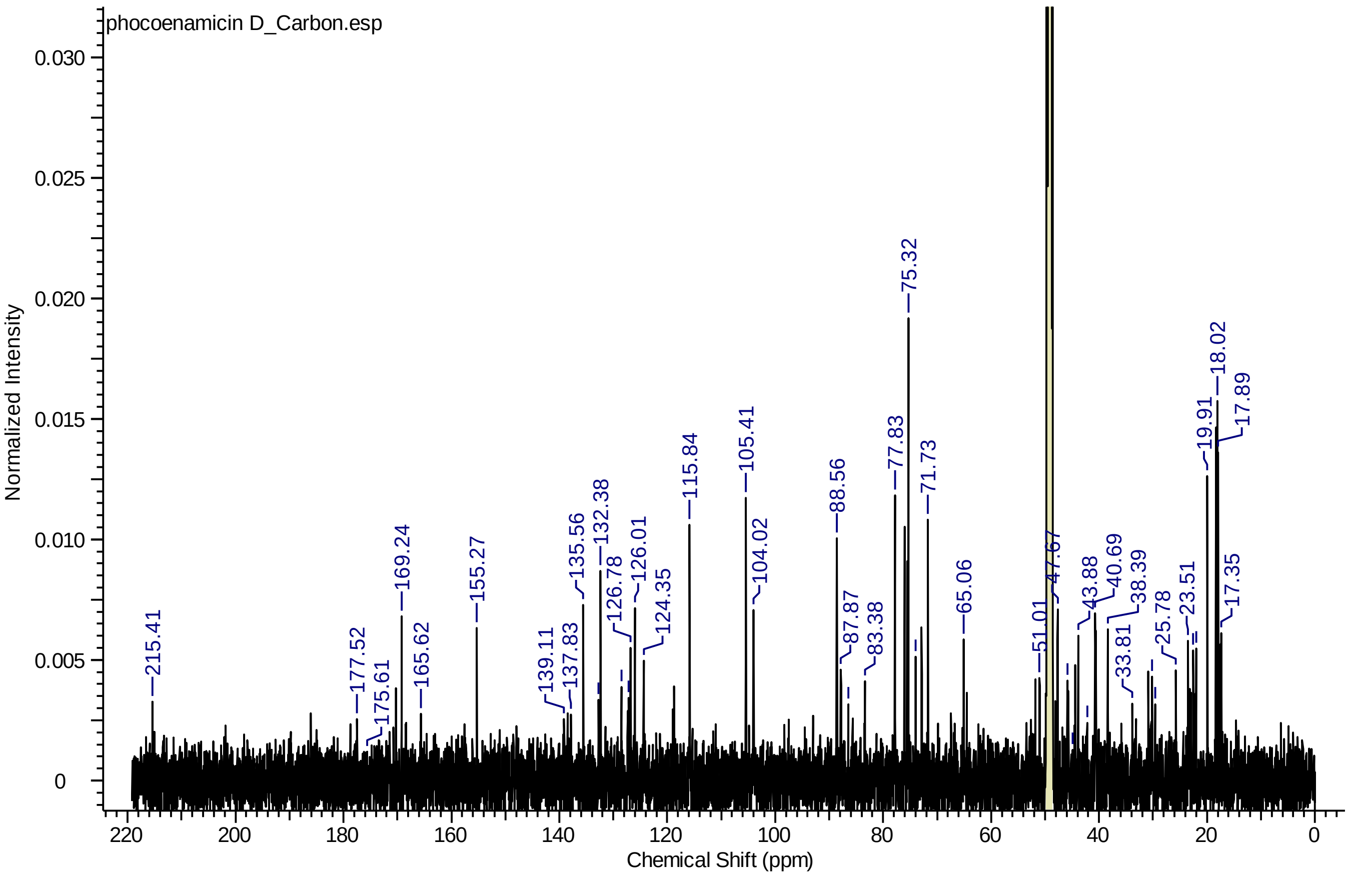

Figure S4. ^{13}C NMR (methanol-d_4, 125 MHz) spectrum of compound **1**.

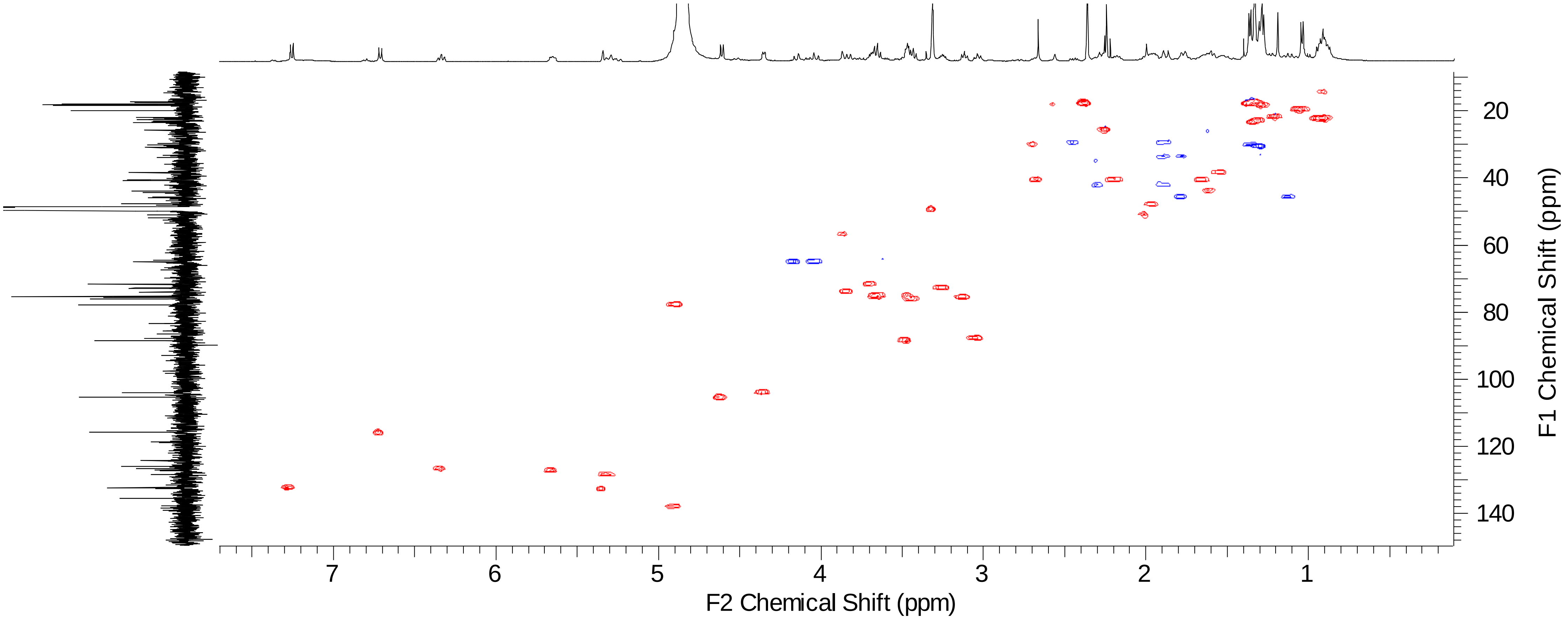

Figure S5. HSQC spectrum of compound **1**.

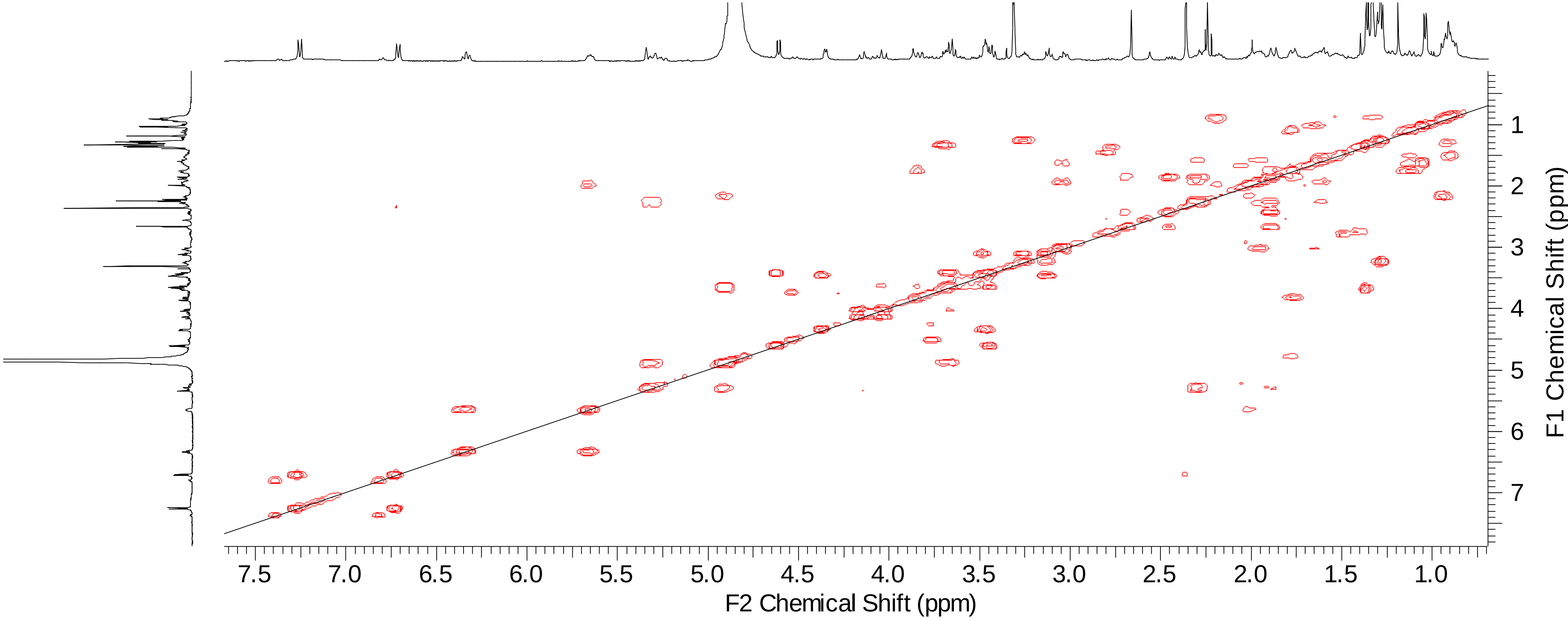

Figure S6. COSY spectrum of compound **1**.

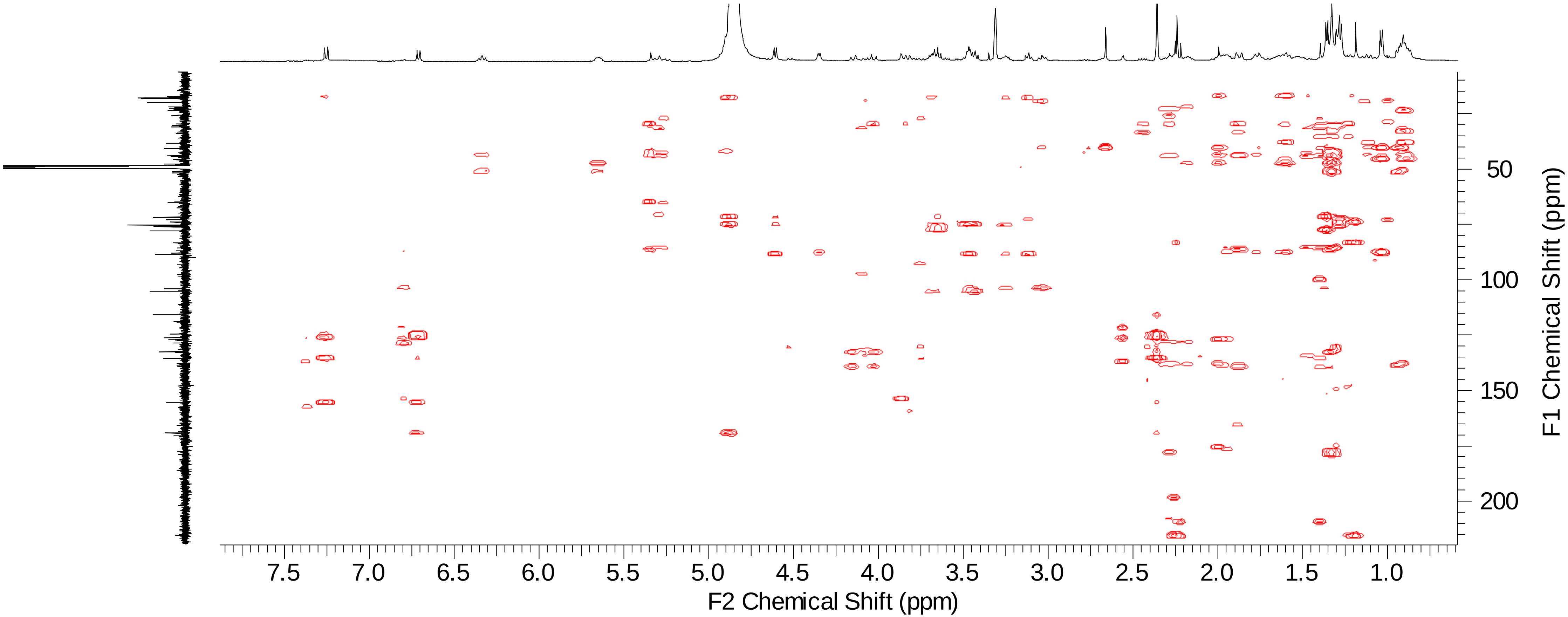

Figure S7. HMBC spectrum of compound **1**.

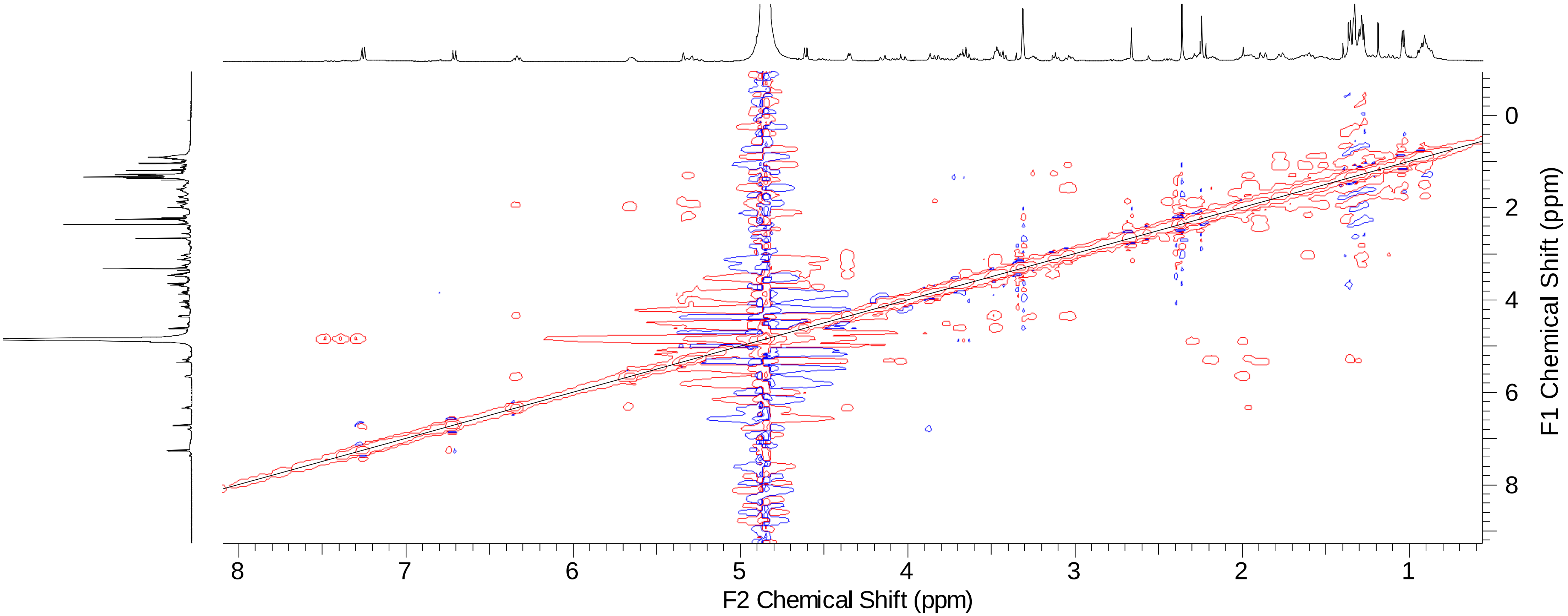

Figure S8. NOESY spectrum of compound **1**.

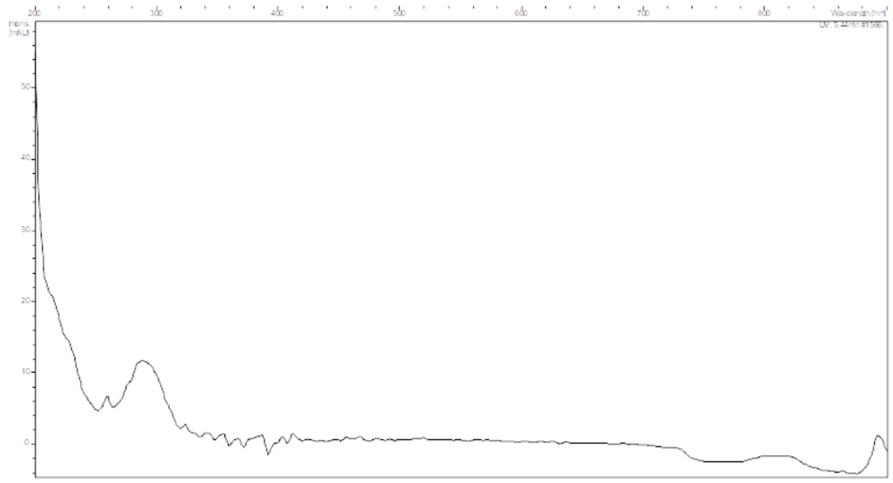

Figure S9. UV spectrum of compound **2**.

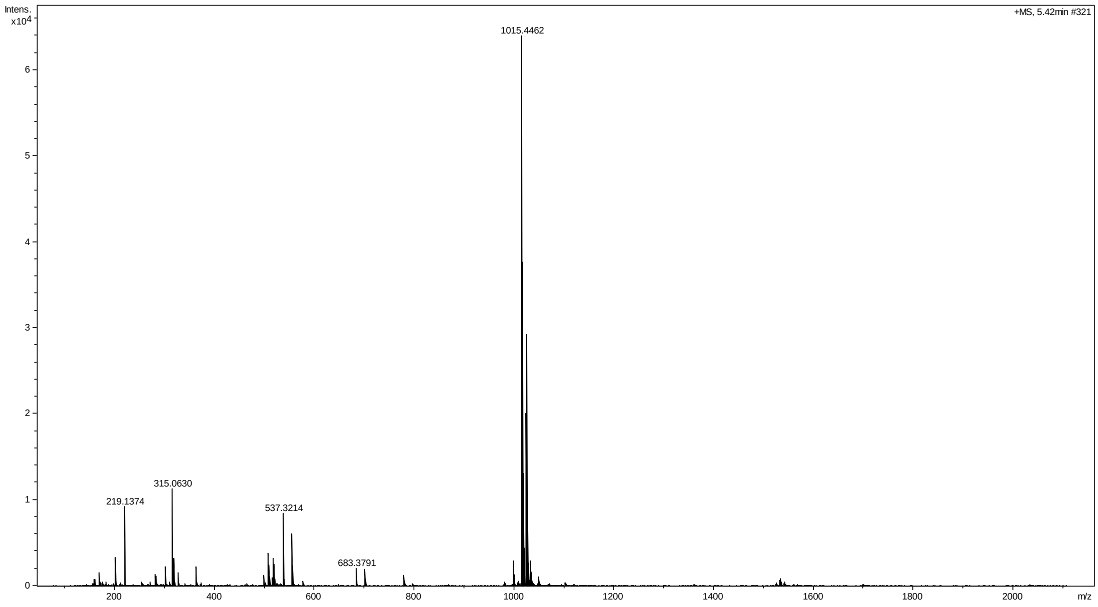

Figure S10. ESI-TOF spectrum of compound **2**.

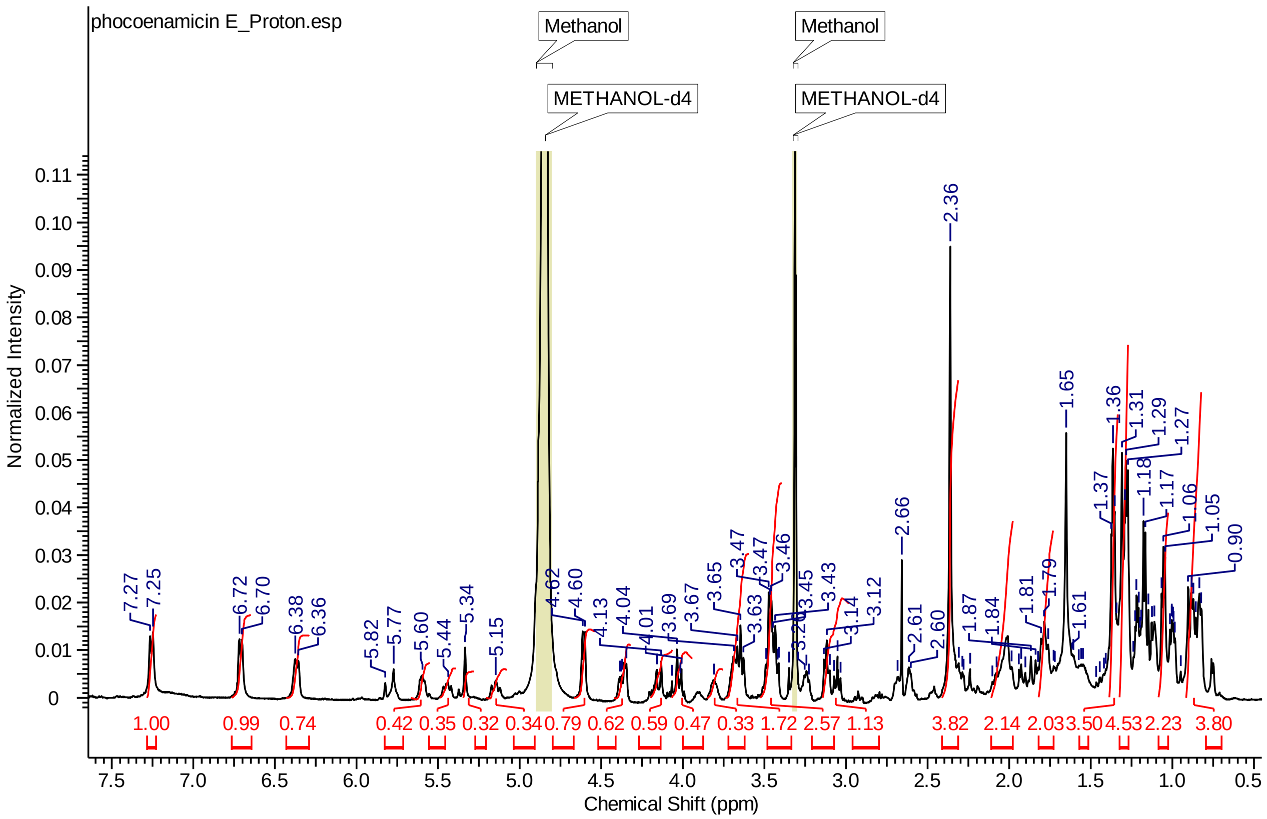

Figure S11. ^{1}H NMR (methanol-d_4, 500 MHz) spectrum of compound **3**.

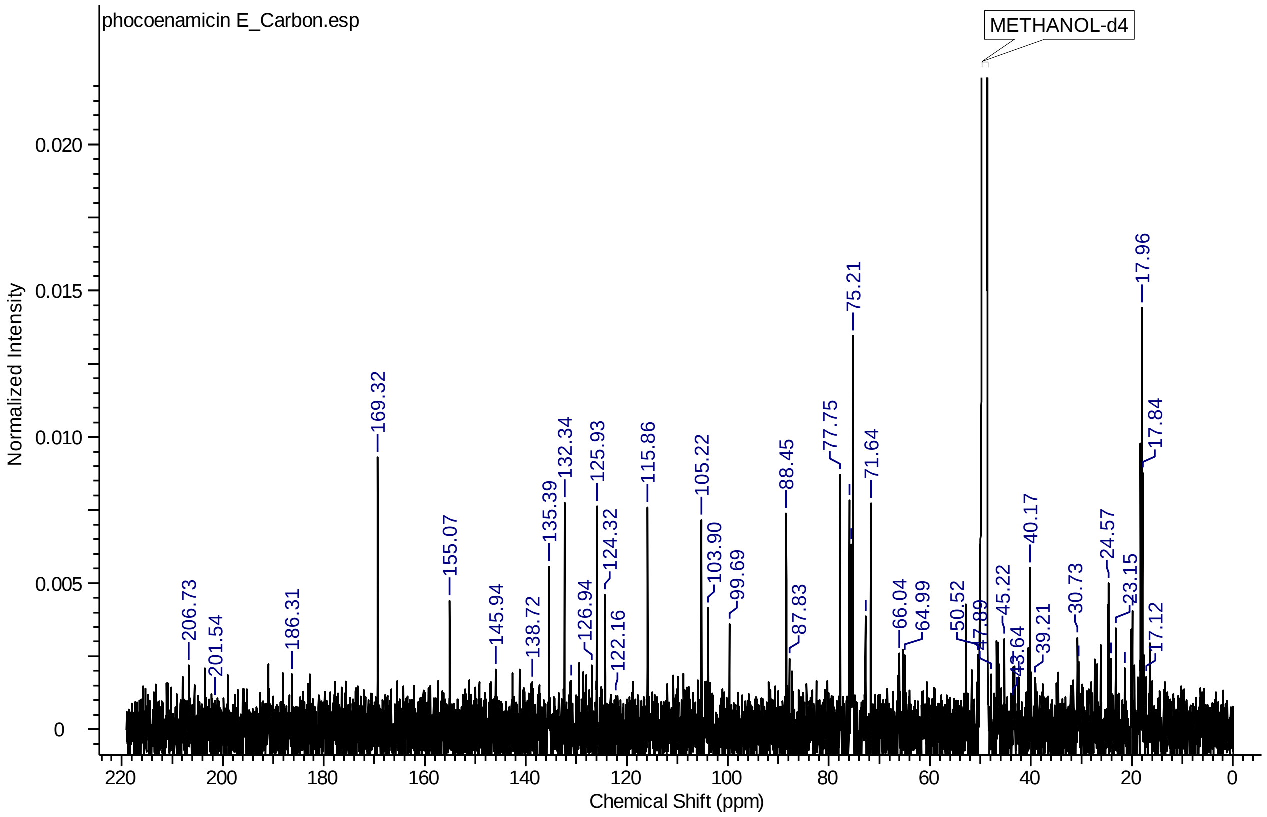

Figure S12. ^{13}C NMR (methanol-d_4, 125 MHz) spectrum of compound **2**.

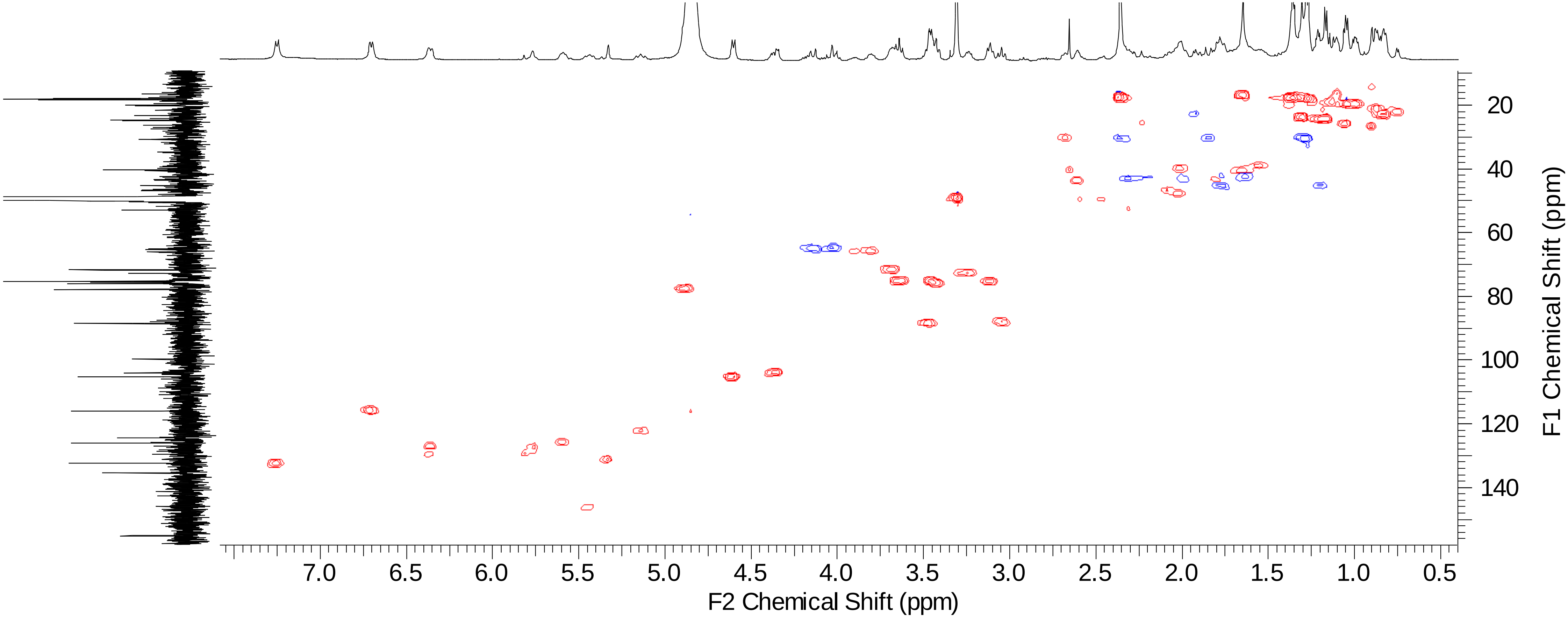

Figure S13. HSQC spectrum of compound **2**.

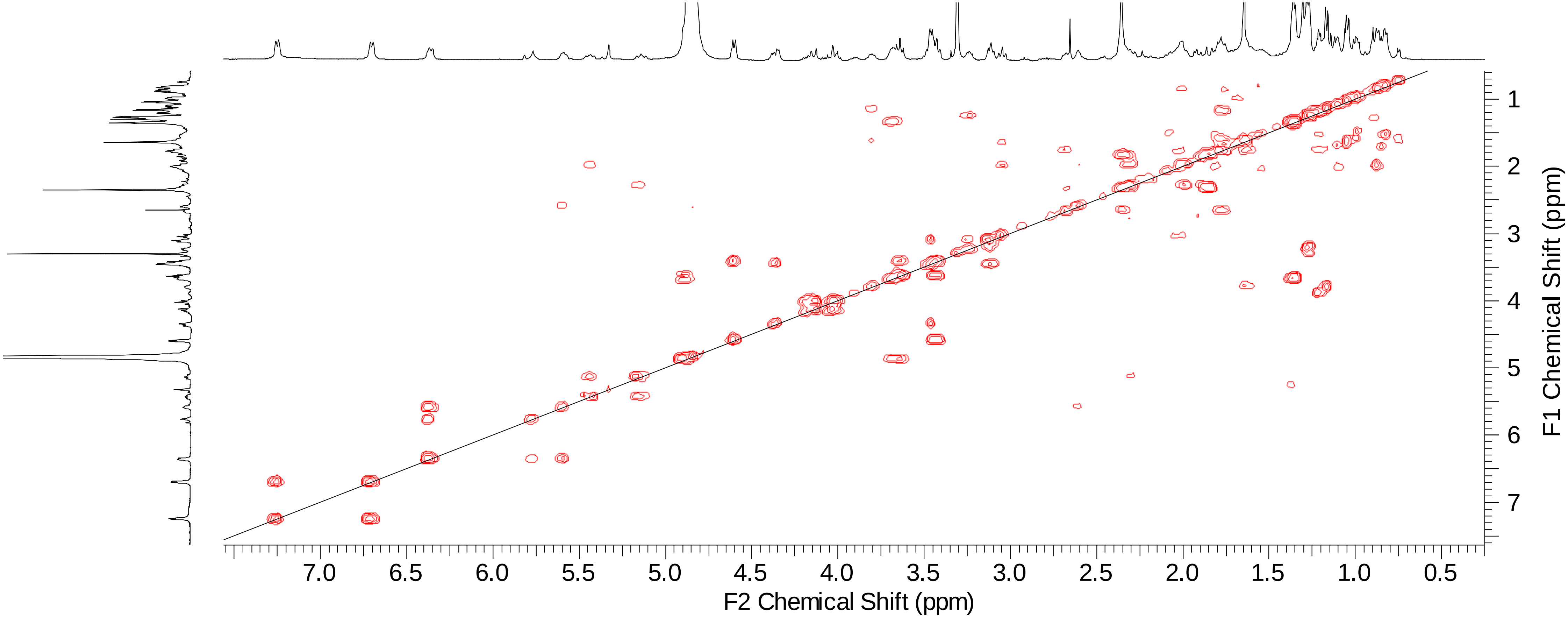

Figure S14. COSY spectrum of compound **2**.

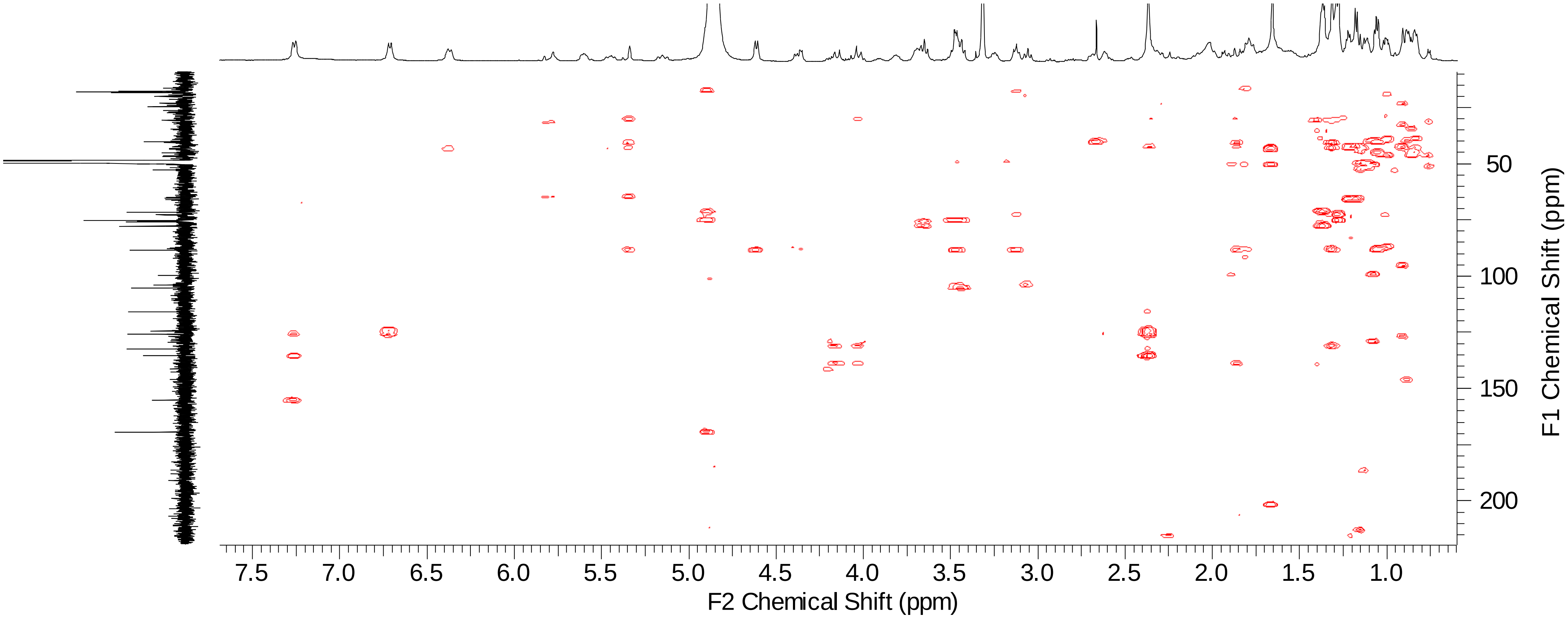

Figure S15. HMBC spectrum of compound **2**.

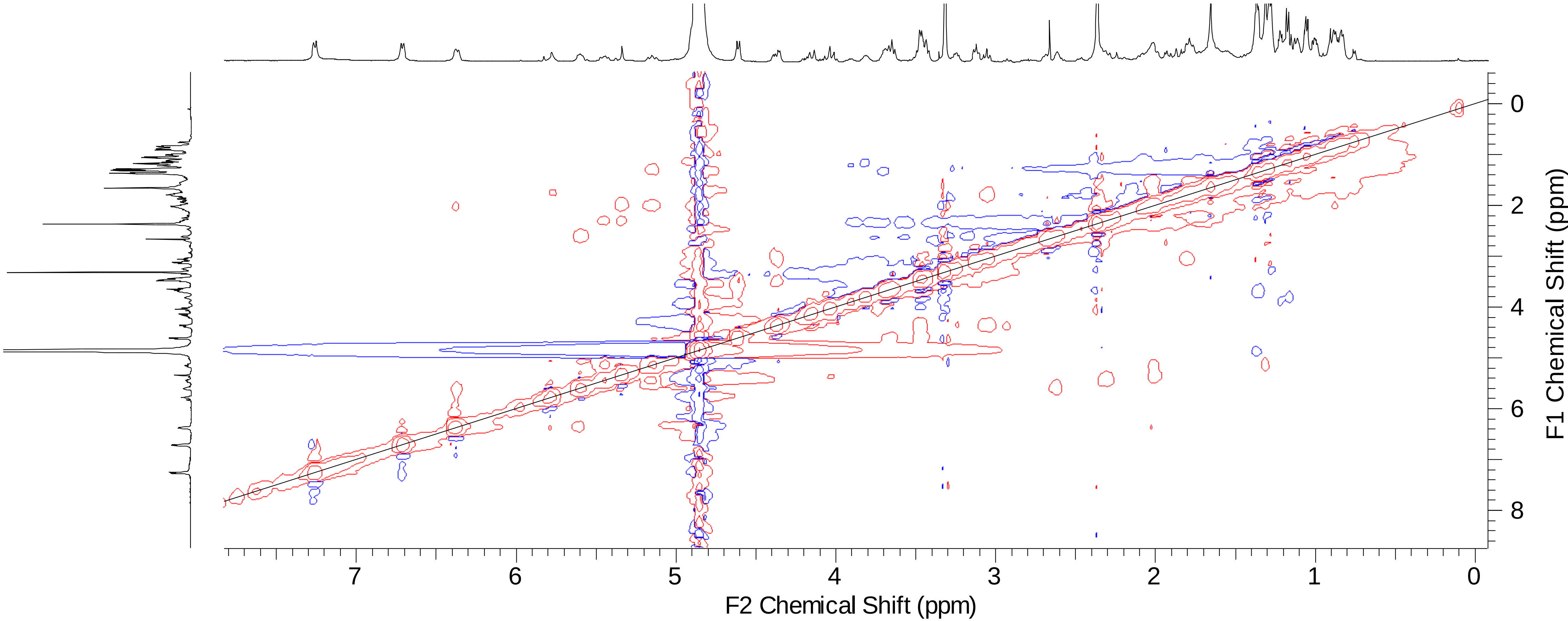

Figure S16. NOESY spectrum of compound **2**.

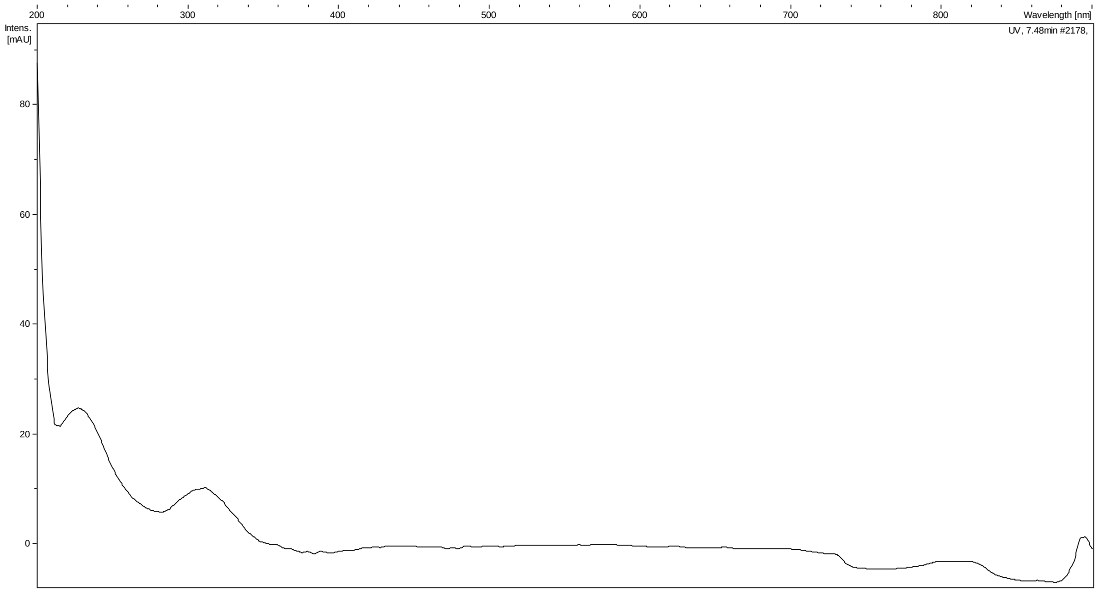

Figure S17. UV spectrum of compound **6**.

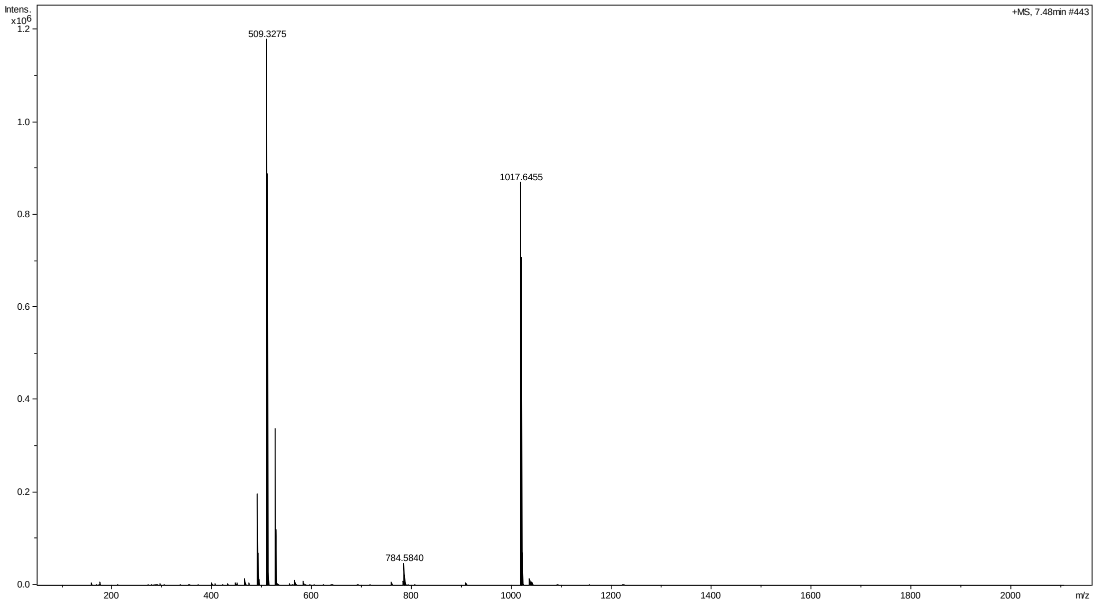

Figure S18. ESI-TOF spectrum of compound **6**.

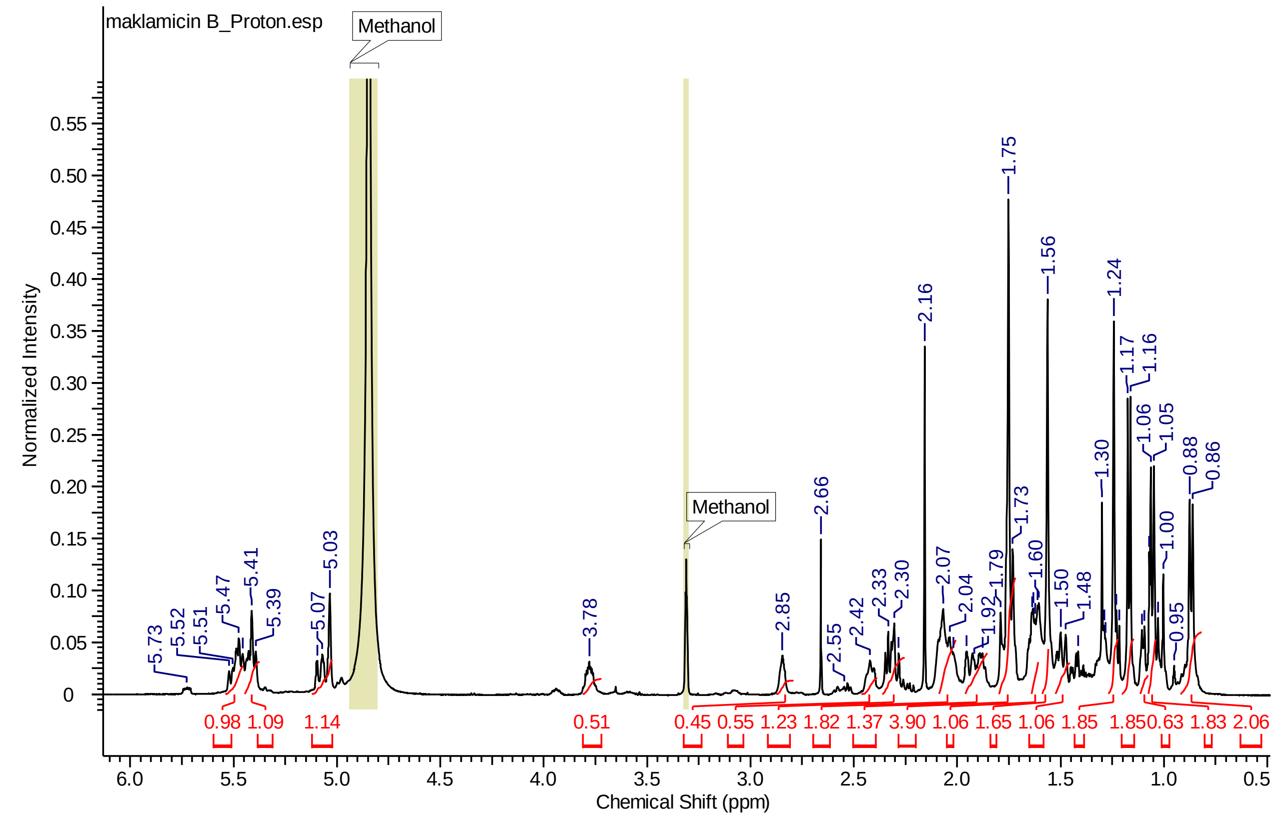

Figure S19. ^{1}H NMR (methanol-*d*$_4$, 500 MHz) spectrum of compound **6**.

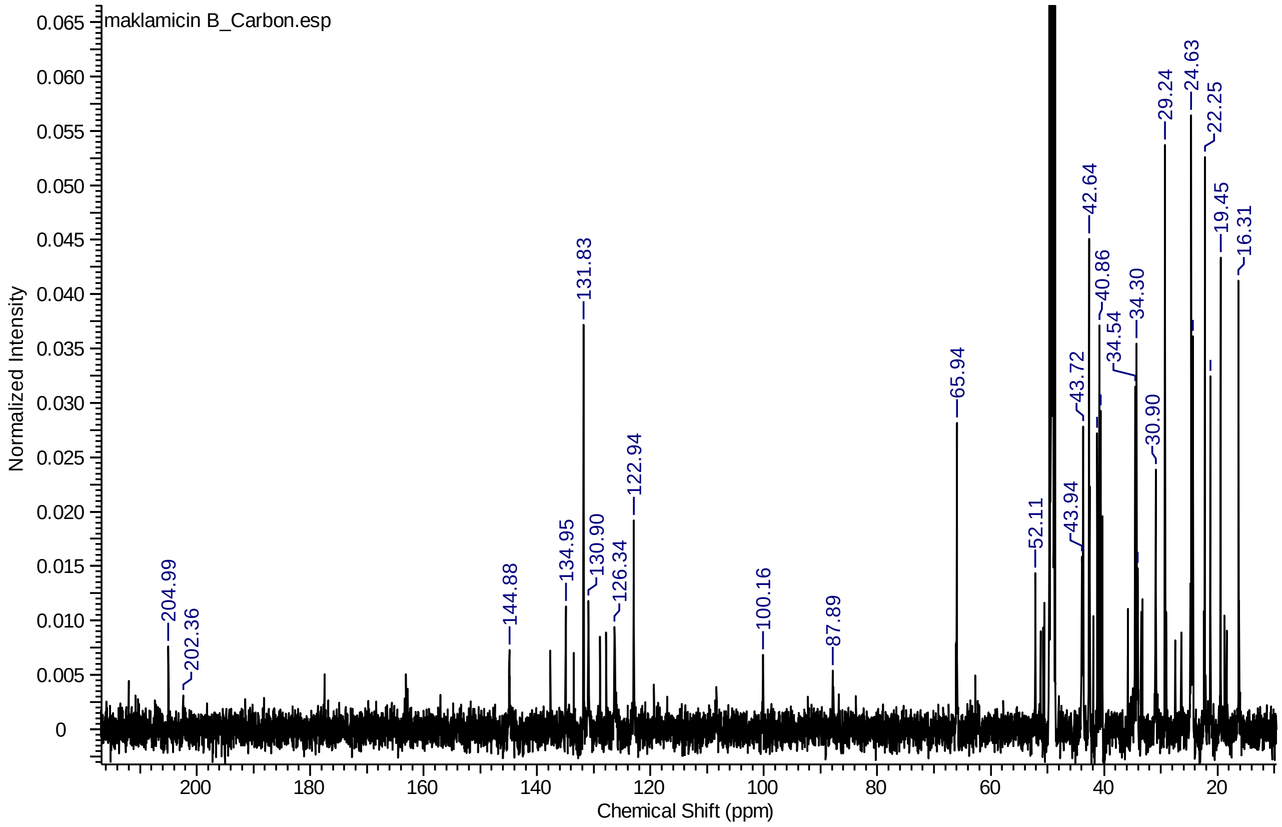

Figure S20. ^{13}C NMR (methanol-*d*$_4$, 125 MHz) spectrum of compound **6**.

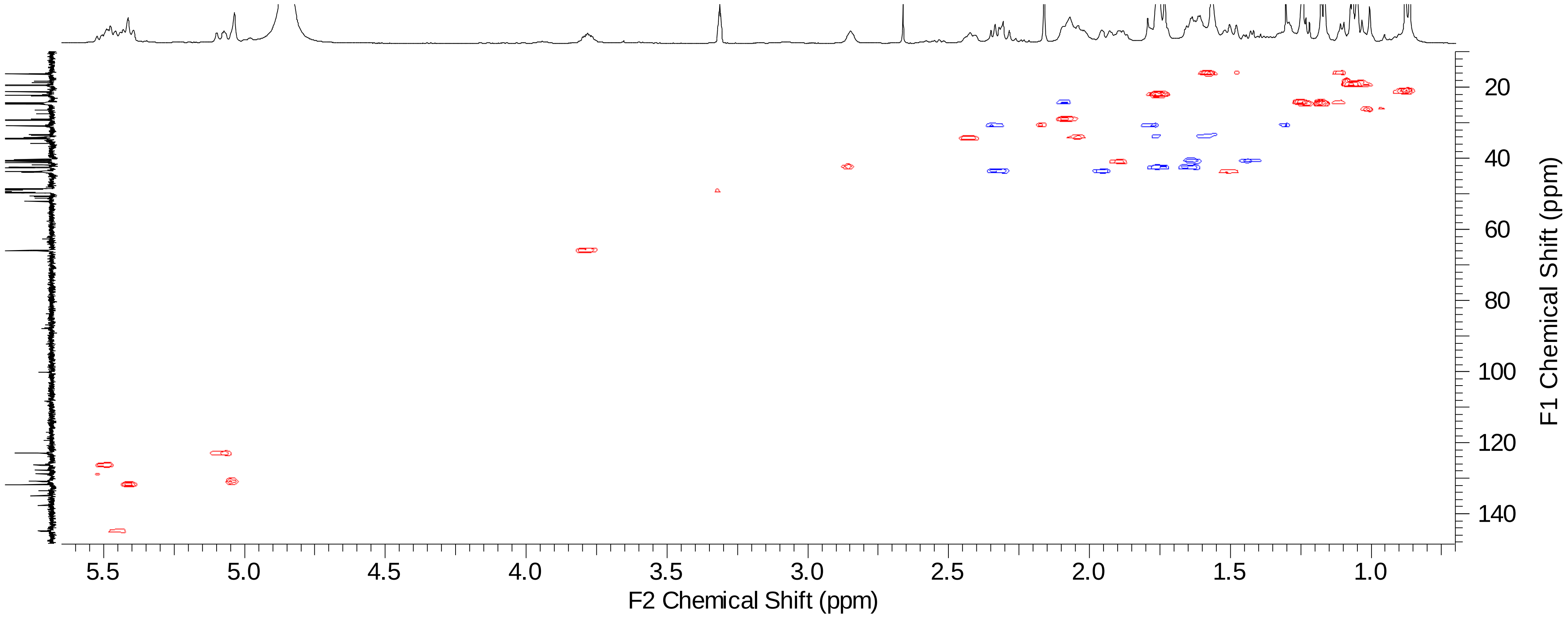

Figure S21. HSQC spectrum of compound **6**.

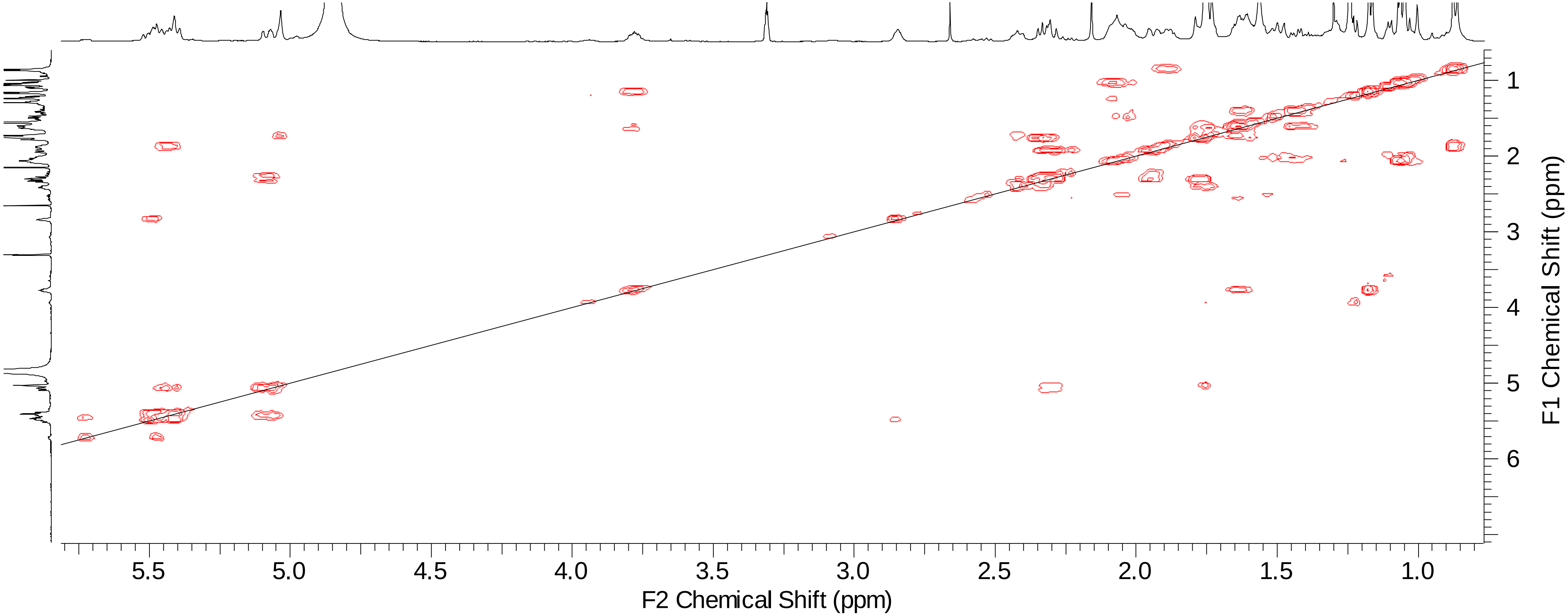

Figure S22. COSY spectrum of compound **6**.

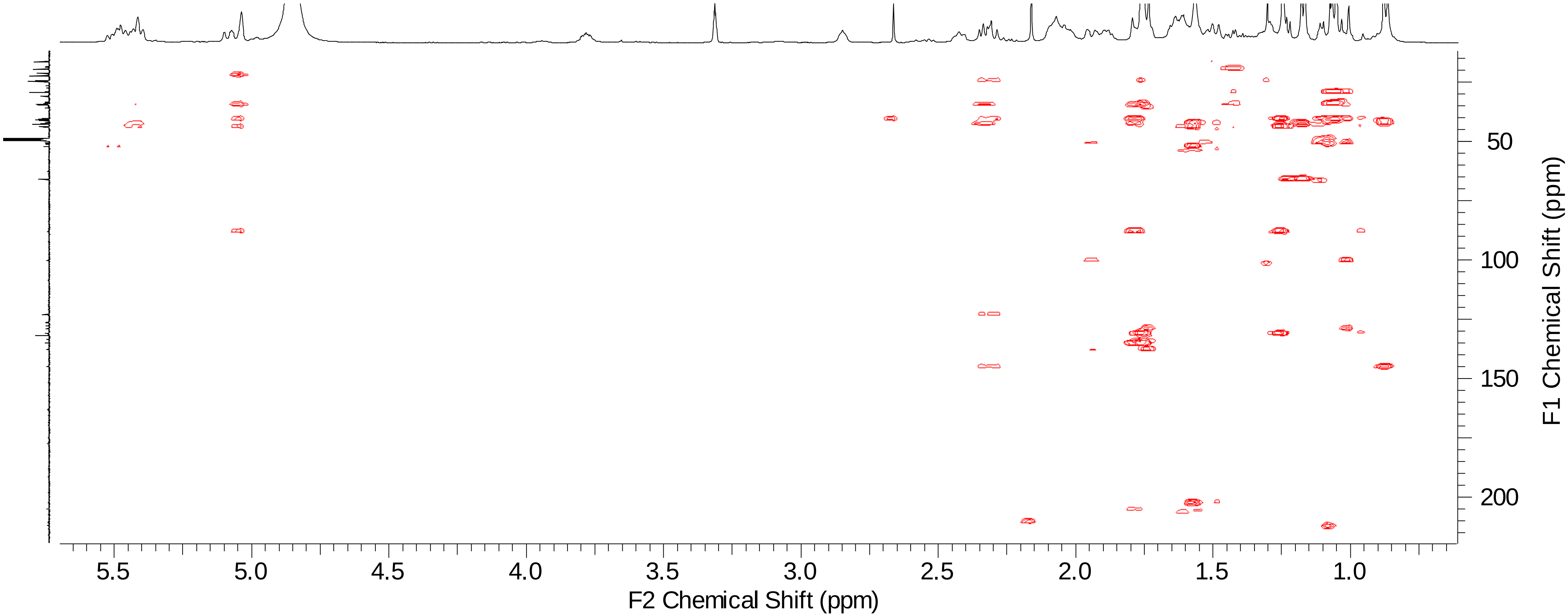

Figure S23. HMBC spectrum of compound **6**.

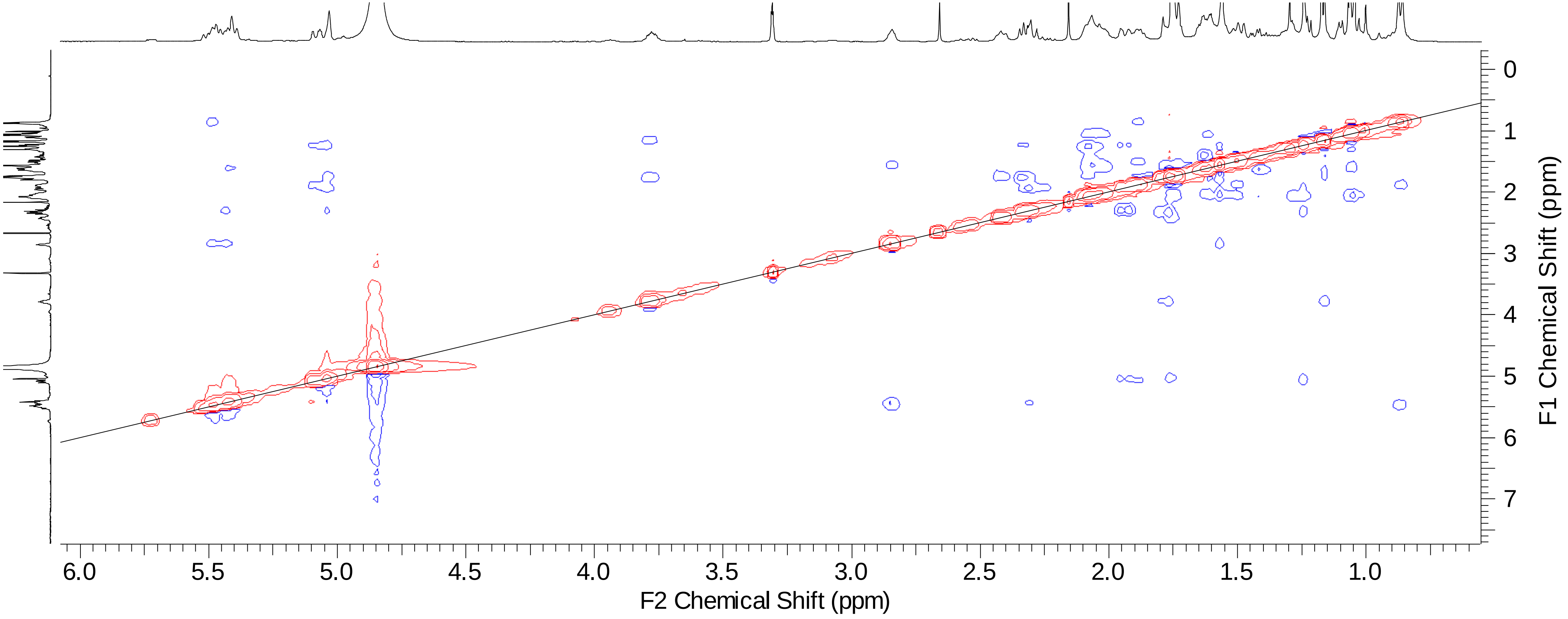

Figure S24. ROESY spectrum of compound **6**.

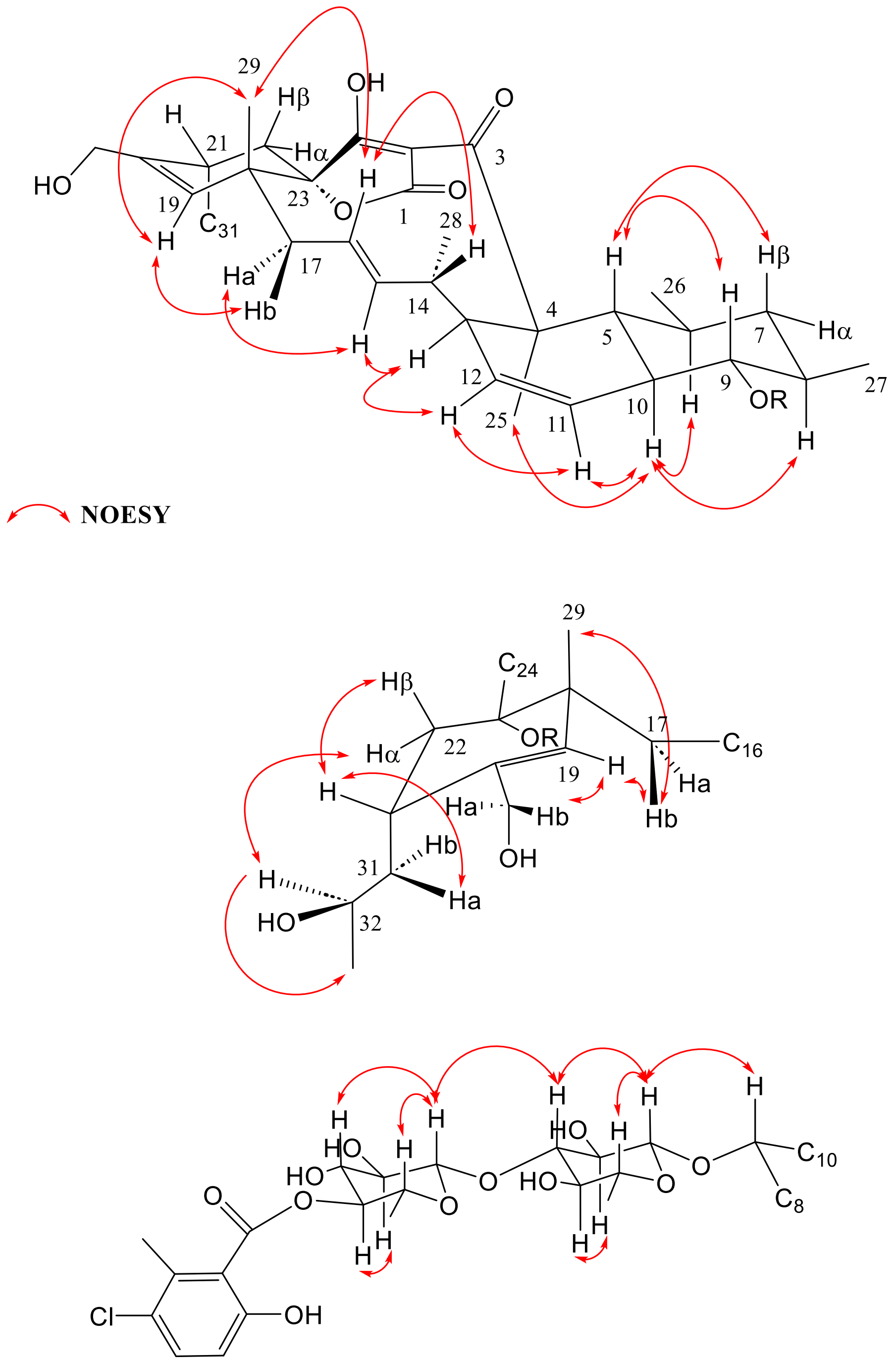

Figure S25. Key NOESY correlations observed in phocoenamicin E (**2**).

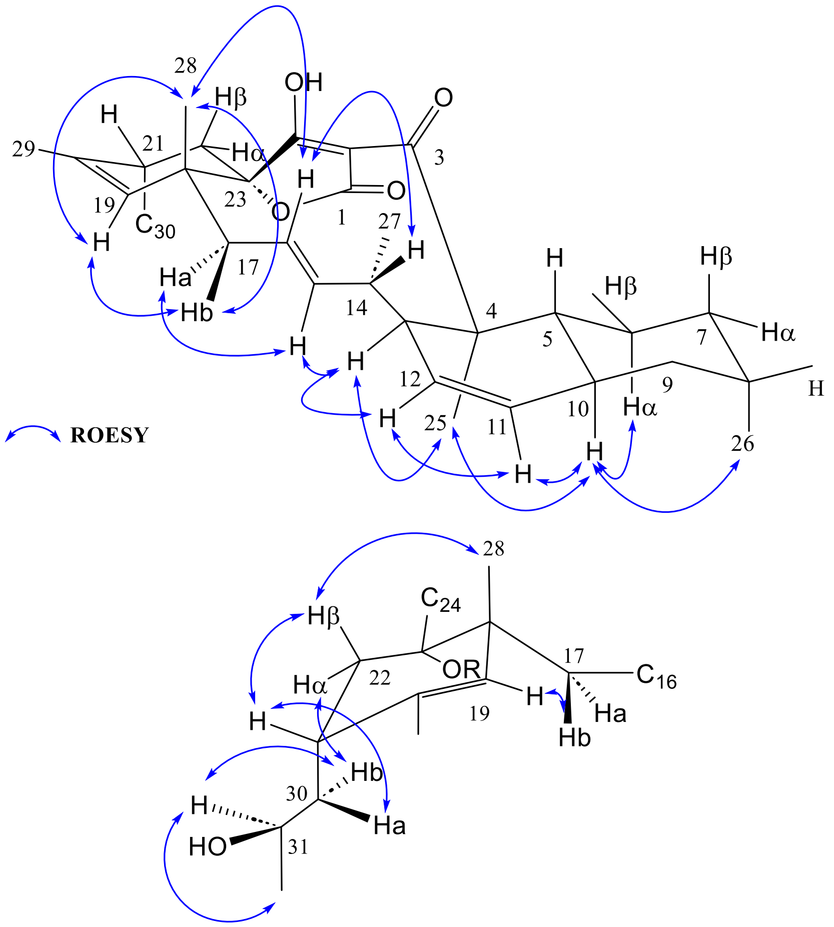

Figure S26. Key ROESY correlations observed in maklamicin B (**6**).
